# Mining of high throughput screening database reveals AP-1 and autophagy pathways as potential targets for COVID-19 therapeutics

**DOI:** 10.1038/s41598-021-86110-8

**Published:** 2021-03-24

**Authors:** Hu Zhu, Catherine Z. Chen, Srilatha Sakamuru, Jinghua Zhao, Deborah K. Ngan, Anton Simeonov, Mathew D. Hall, Menghang Xia, Wei Zheng, Ruili Huang

**Affiliations:** Division of Preclinical Innovation, National Center for Advancing Translational Sciences (NCATS), National Institutes of Health (NIH), DPI/NCATS, 9800 Medical Center Drive, Rockville, MD 20850 USA

**Keywords:** Target identification, High-throughput screening, Data mining

## Abstract

The recent global pandemic of the Coronavirus disease 2019 (COVID-19) caused by the new coronavirus SARS-CoV-2 presents an urgent need for the development of new therapeutic candidates. Many efforts have been devoted to screening existing drug libraries with the hope to repurpose approved drugs as potential treatments for COVID-19. However, the antiviral mechanisms of action of the drugs found active in these phenotypic screens remain largely unknown. In an effort to deconvolute the viral targets in pursuit of more effective anti-COVID-19 drug development, we mined our in-house database of approved drug screens against 994 assays and compared their activity profiles with the drug activity profile in a cytopathic effect (CPE) assay of SARS-CoV-2. We found that the autophagy and AP-1 signaling pathway activity profiles are significantly correlated with the anti-SARS-CoV-2 activity profile. In addition, a class of neurology/psychiatry drugs was found to be significantly enriched with anti-SARS-CoV-2 activity. Taken together, these results provide new insights into SARS-CoV-2 infection and potential targets for COVID-19 therapeutics, which can be further validated by in vivo animal studies and human clinical trials.

## Introduction

In late fall 2019, a new Coronavirus disease 2019 (COVID-19) emerged from Wuhan, China. It is caused by the severe acute respiratory syndrome coronavirus 2 (SARS-CoV-2). SARS-CoV-2 appears to be highly contagious, and a lack of immunity in the human population has resulted in rapid spread across the globe. As of November 2020, the virus has infected over 50 million people, killed over 1.3 million people, and caused abrupt disruption to social and economic activities around the world (https://covid19.who.int/).

The treatment for COVID-19 evolved rapidly in early 2020 after its emergence, from supportive care (supplemental oxygen^1^), nonspecific therapies (dexamethasone^2^, convalescent plasma^3^), to specific therapies (Bamlanivimab^4^). For preventive approaches such as vaccines, Pfizer announced the first compelling evidence that a vaccine can prevent COVID-19, which was more than 90% effective at preventing disease, on November 9th, 2020^5^. More recently on November 16, 2020, Moderna released preliminary data on another vaccine that was found to be more than 94% effective at preventing severe COVID-19 infection^6^. Typically, small molecule drug development takes 12–16 years and costs US$1–2 billion to bring a new drug to the market^7^. Given that treatments for patients infected with SARS-CoV-2 are needed immediately, repurposing existing drugs and clinical investigational drugs to treat COVID-19 is an attractive strategy. This approach takes advantage of known human pharmacokinetics and safety profiles of drugs, which allows for rapid initiation of human clinical trials or direct use for treatments. Remdesivir is such an example of repurposing an existing drug to treat COVID-19. In a double-blind, randomized, placebo-controlled trial carried out by the National Institutes of Health (NIH), remdesivir was demonstrated to be effective in reducing the recovery time from 15 to 11 days in hospitalized COVID-19 patients^8^. On May 1, 2020, the U.S. Food and Drug Administration (FDA) issued an emergency use authorization (EUA) for the investigational antiviral drug remdesivir for the treatment of hospitalized COVID-19 patients. However, the beneficial effect of remdesivir was challenged by the most recent results from the World Health Organization (WHO)^9^.

Rather than taking an intuitive repurposing approach based on known mechanisms (as demonstrated by the recent reports on beneficial effects of dexamethasone for modulating inflammatory response in COVID-19)^10^, an unbiased and systematic screening of approved or clinical investigational drugs may uncover additional therapeutic options. Multiple sites^11–15^, including our center (The National Center for Advancing Translational Sciences, NCATS), are screening approved drugs and mechanistically annotated libraries to identify new therapeutics. To rapidly share screening results with the scientific community and accelerate the drug repurposing process, NCATS created a freely available, online database that contains a collection of COVID-19-related drug repurposing screening data for all approved drugs as well as the assay protocols used to generate them(Open Science Data Portal of COVID-19) (https://opendata.ncats.nih.gov/covid19/index.html)^16^. In most antiviral drug repurposing efforts, the most scalable assay used for screening in biological safety level-3 laboratories is a phenotypic assay, which measures the cytopathic effect (CPE) of SARS-CoV-2 virus on Vero E6 cells infected for 72 h. If compounds exhibit antiviral activity, Vero E6 cells are rescued from the CPE. While many drugs have known targets/mechanisms of action for their approved indications, the targets or mechanisms of their antiviral activities remain largely unknown, which could be a host of viral targets^11–15^. It is thus crucial to better understand the antiviral mechanisms of these drugs to facilitate further drug development.

The NCATS Pharmaceutical Collection (NPC)^17^ is a library of ~ 3,000 drugs approved for marketing in the US (FDA), Europe (EMA), Canada, Australia, and/or Japan (PMDA). The library was specifically created to enable drug repurposing and it has been screened at NCATS in nearly 1,000 assays in concentration–response (quantitative high throughput screening, qHTS). These assays encompass a wide range of disease targets and pathways with main disease areas covered including rare and neglected diseases, infectious diseases, and cancer^18^. Here, we leveraged this unique dataset to compare activity across SARS-CoV-2 CPE screening data (both from NCATS and published elsewhere)^15,19–24^ with historical in-house NPC qHTS data. Correlations were performed to identify assays with patterns of activity similar to that shown in the SARS-CoV-2 CPE assay.

## Results

### Mining qHTS data reveals potential anti-SARS-CoV-2 targets: Autophagy and AP-1 signaling

**Figure 1:**
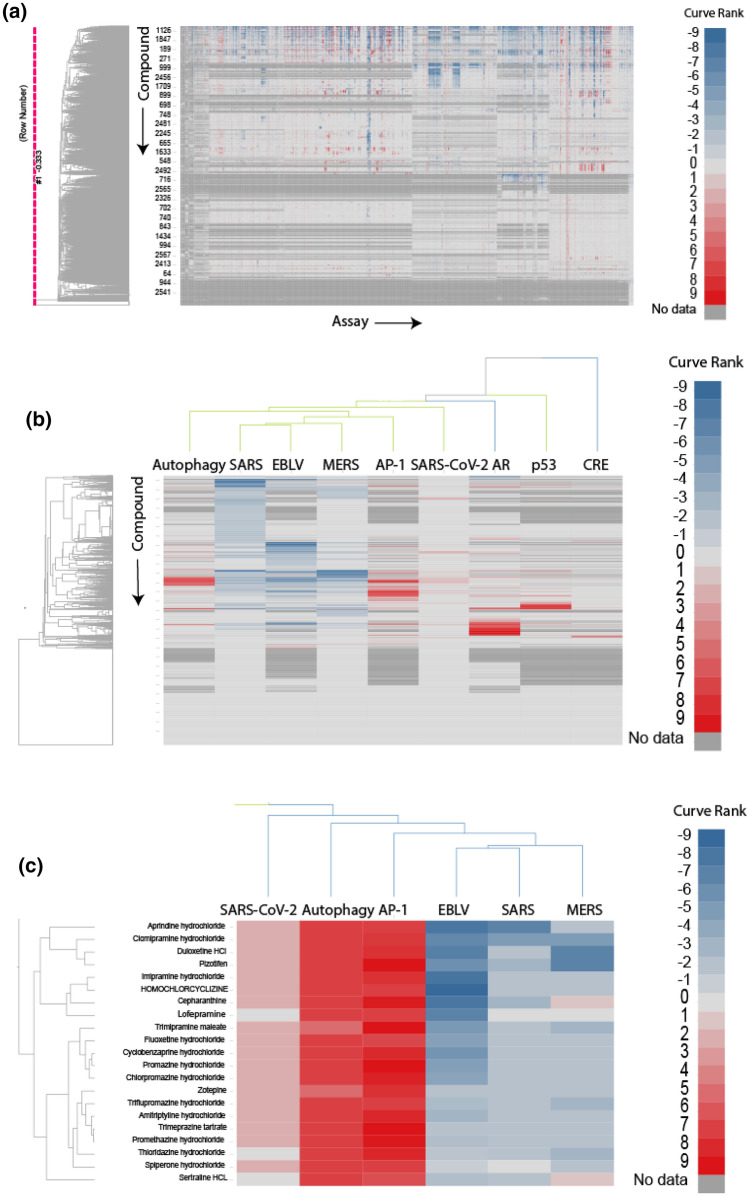
Compound activity profiles from the ~ 1000 NPC screens. In the heat map, each row is a compound, and each column is an assay. The heat map is colored by “curve rank”^26,27^, a numeric measure (between -9 and 9) of compound activity based on potency, efficacy, and the quality of the concentration response curve, such that a large positive number indicates a strong activator (red), i.e., a compound that activates or induces the assay target activity, a large negative number indicates a strong inhibitor (blue), i.e., a compound that inhibits the assay target activity, and 0 means inactive (light gray). Dark gray indicates missing data. (**a**) All assays. (**b**) Examples of assays that showed significant correlations with the SARS-CoV-2 CPE assay (SARS-CoV, MERS, EBLV, autophagy, AP-1) and assays that showed no correlation with the SARS-CoV-2 CPE assay (p53; *AR* androgen receptor; *CRE* cAMP response element). (**c**) A zoomed-in view that shows the activities of a group of mostly psychoactive drugs that were active in the SARS-CoV-2 CPE assay as well as the autophagy and/or AP-1 assays.

**Figure 2:**
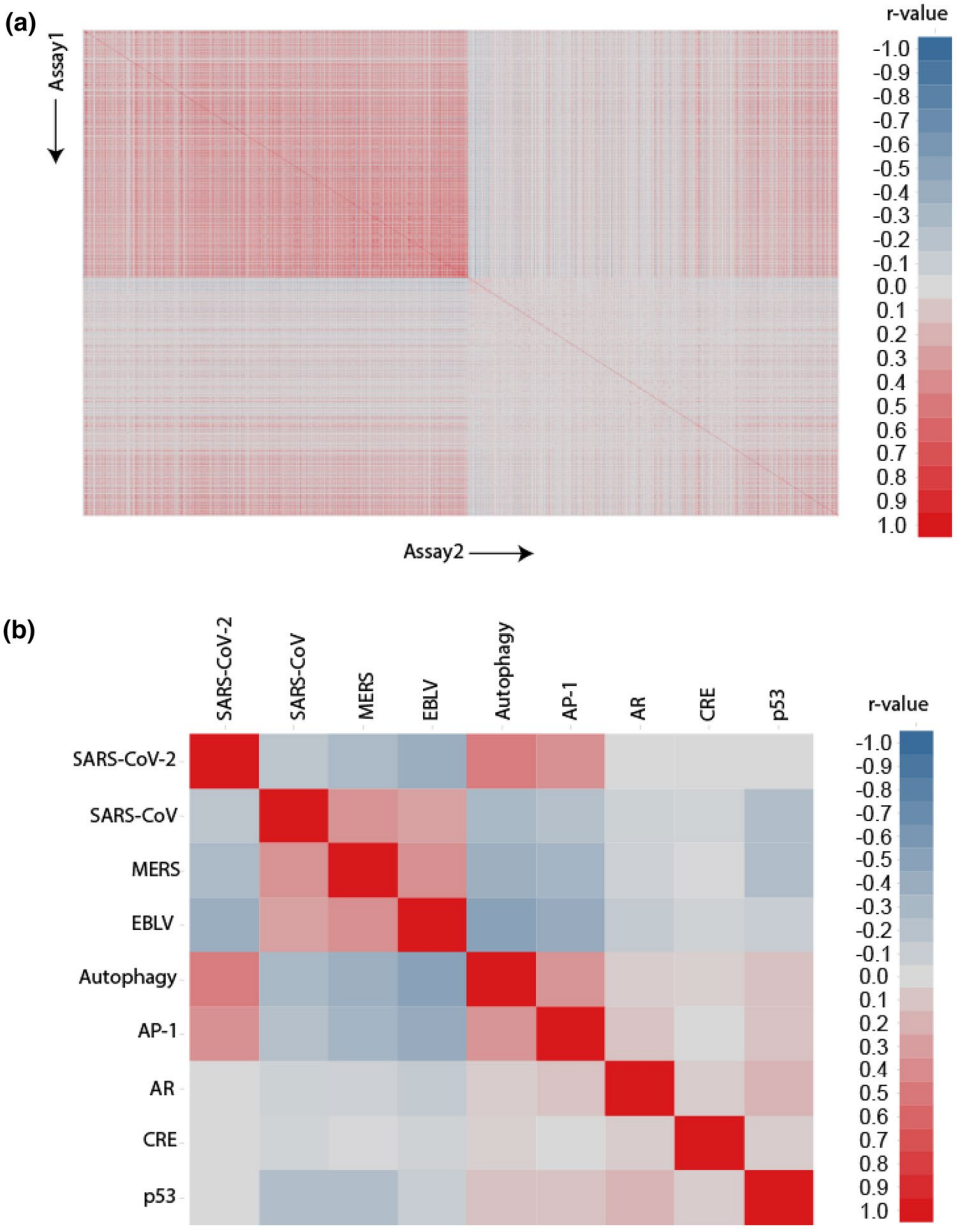
Activity profile correlations between the ~ 1000 NPC screens including the SARS-CoV-2 CPE assay. In the heat map, each row/column is a different assay. The heat map is colored by the correlation coefficient (*r*), such that darker shades of red indicate stronger positive correlations and darker shades of blue indicate stronger negative correlations. Gray means no correlation found. (**a**) Pair-wise correlations of all assays. (**b**) A zoomed-in view that shows the pair-wise correlations of example assays that either significantly correlated or showed no correlation with the SARS-CoV-2 CPE assay.

Phenotypic assays, such as the CPE assay, have been used to screen compound libraries to identify compounds that prevented cell death caused by SARS-CoV-2 infection. Assays that share similar activity profiles, i.e., similar active and inactive compounds, may have molecular targets in common. In order to identify new anti-SARS-CoV-2 targets, we compared the compound activity profiles^18^ in each of the ~ 1000 NPC screens with the activity profiles in the SARS-CoV-2 CPE assay. The assays with activity profiles which resemble that of the SARS-CoV-2 CPE assay may represent targets or pathways related to SARS-CoV-2. These targets or pathways could provide important clues to the underlying mechanisms of the pathogenesis of SARS-CoV-2, and serve as targets for the development of new COVID-19 therapies. Toward this goal, we gathered compounds reported as active from recent anti-COVID-19 repurposing screens in the literature using the SARS-CoV-2 CPE assay (including the NPC screen results)^15,19,20,25^ and drugs proposed by the scientific community as potential COVID-19 therapies^11,21,23,24^. A list of these compounds is provided in Supplementary Table 1. Activities of these compounds in the CPE assay were used as a “probe signature” to compare with the activity profiles of the ~ 1000 NPC screens (Fig. 1a). Compound activity was represented by “curve rank”,^26,27^ a numeric measure between -9 and 9 based on potency, efficacy, and the quality of the concentration response curve, such that a large positive number indicates a strong activator, a large negative number indicates a strong inhibitor, and 0 means inactive. Activity profile similarity was measured by the Pearson Correlation Coefficient (*r*) with a p-value calculated for the significance of correlation (Fig. 2).

**Table 1:** Activity concordance between the SARS-CoV-2 CPE assay and top correlated assays.

	Autophagy	AP-1	Autophagy + AP-1
TP	88	84	118
FN	44	29	21
FP	142	399	473
TN	1887	1243	1682
Sensitivity	0.67	0.74	0.85
Specificity	0.93	0.76	0.78
BA	0.80	0.75	0.81

TP True positive; number of compounds active in both the SARS-CoV-2 and the other assay. *FN* false negative; number of compounds active in the SARS-CoV-2 assay but not active in the other assay. *FP* false positive; number of compounds not active in the SARS-CoV-2 assay but active in the other assay. TN = True negative; number of compounds inactive in both assays. *BA* balanced accuracy; (sensitivity + specificity)/2.

Sensitivity = TP/(TP + FN).

Specificity = TN/(TN + FP).

The activity profiles from an autophagy assay (*r* = 0.47, *P* < 1 × 10^–20^)^28^ and an AP-1 signaling pathway assay (*r* = 0.37, *P* < 1 × 10^–20^)^29^ exhibited the most significant correlations with that of the SARS-CoV-2 screen (Fig. 1b). Interestingly, three other antiviral assays, an Ebola virus-like particle entry assay (EBOV) (*r* = 0.39, *P* < 1 × 10^–20^),^30^ a MERS pseudo particle entry assay (*r* = 0.28, *P* < 1 × 10^–20^)^31^ and a SARS-CoV pseudo particle entry assay (*r* = 0.18, *P* = 2.77 × 10^–20^)^31^, were also among the most significantly correlated assays with activity profiles that highly resemble that of the SARS-CoV-2 CPE assay (Fig. 1b). As SARS-CoV is the closet relative to SARS-CoV-2 and MERS belongs to the same family of beta-coronaviruses, these findings can serve as a validation of our approach.^32–34^ Moreover, remdesivir, an antiviral drug active against Ebola, was recently found to be effective against SARS-CoV-2 in in vitro assays and approved for treating hospitalized COVID-19 patients^8,35^. This is consistent with our finding that a significant number (118) of drugs including remdesivir demonstrated both anti-Ebola activity and activity in the SARS-CoV-2 CPE assay, suggesting the presence of shared drug targets (either viral target or cellular targets) between EBOV and SARS-CoV-2. The activity profiles of three pathway assays, androgen receptor (AR) signaling pathway^36^, cAMP response element (CRE) signaling pathway^37^, and p53 signaling^38^, serve as examples of assays that exhibited no correlation with the SARS-CoV-2 CPE assay (*r* = 0), and these are shown in Fig. 1b for comparison purposes.

To further examine the compound activity concordance between SARS-CoV-2 and the top correlated assays, we investigated if the compound profiles of each assay could be used as predictors for the activity in the SARS-CoV-2 CPE assay (Table 1). Compound profiles in both the autophagy and the AP-1 assays showed good concordance with that in the SARS-CoV-2 CPE assay with balanced accuracies (BA) of 0.80 and 0.75, respectively. The autophagy assay showed very high specificity (0.93) and relatively low sensitivity (0.65) in picking up the SARS-CoV-2 actives, whereas the AP-1 assay showed slightly higher specificity (0.76) compared to sensitivity (0.74). When the autophagy and AP-1 assays were combined, i.e., a compound was counted as active if it was active in either one of these assays and inactive otherwise, the sensitivity in picking up SARS-CoV-2 actives increased to 0.85 with an improved BA of 0.81. The improvement in sensitivity and BA suggests that the targets in these two assays are different and either autophagy or AP-1 could only account for one mechanism in targeting SARS-CoV-2. The combination of the two pathways may increase the likelihood of identifying drugs that can target SARS-CoV-2 through different mechanisms.

**Figure 3:**
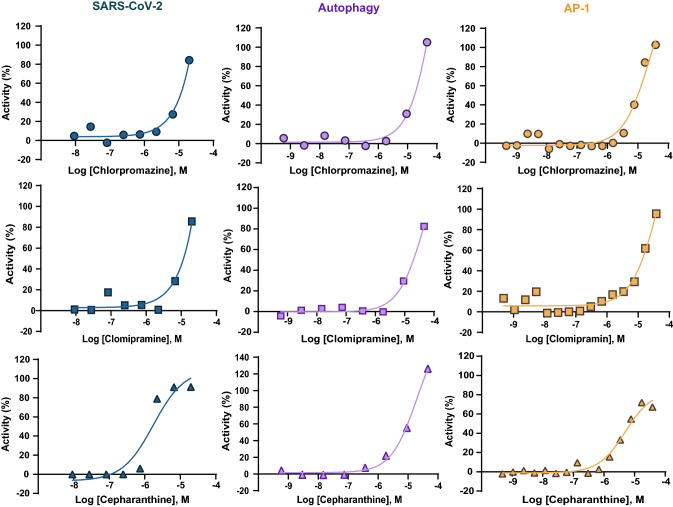
Example concentration–response curves of compounds active in the SARS-CoV-2, autophagy, and AP-1 assays. Chlorpromazine is a tricyclic antipsychotic, clomipramine is a tricyclic antidepressant, and cepharanthine is a natural product anti-inflammatory that is approved in Japan^66^.

A list of the most potent compounds (< 20 µM) in the AP-1 assay and their corresponding activities in the autophagy and SARS-CoV-2 CPE assays are provided in Table 2. These drugs can be considered for further anti-COVID-19 development. Concentration–response curves of exemplar compounds that were active in all three assays are shown in Fig. 3.

**Table 2:** Potent (< 20 µM) AP-1 compounds that were active in the SARS-CoV-2 CPE assay or reported as active in the literature.

Compound Name	AP-1 Potency (µM)	Autophagy Potency (µM)	SARS-CoV-2 CPE Potency (µM)	Literature Reported anti-SARS-CoV-2	Neurology/psychiatry
Oxyphenisatin	0.22	N/A	N/A	Y	
Clioquinol	0.37	> 100	10.00		
Trimipramine maleate	1.34	9.02	10.00		Y
Promethazine	2.05	26.60	12.59	Y	Y
Dimethisoquin	2.49	10.12	12.59		Y
Cepharanthine	3.79	13.33	2.00		
Pizotifen	4.25	16.79	12.59		Y
Ethopropazine	4.25	> 100	12.59		Y
Amitriptyline	5.29	10.59	12.59		Y
Mefloquine	5.78	29.85	> 100	Y	
Tolterodine Tartrate	6.49	14.96	12.59	Y	
Tetraethylthiuram disulfide	6.66	2.54	> 100	Y	Y
Lynestrenol	7.14	> 100	12.59		
Cyproheptadine	7.28	> 100	3.98		
Benzydamine	7.28	> 100	12.59		
Promazine	7.56	5.69	10.00	Y	Y
Triparanol	7.56	21.13	N/A	Y	
Fluphenazine	7.56	13.33	> 100	Y	Y
Imipramine	7.76	13.33	8.91		Y
Loxapine succinate	7.86	14.30	10.00		Y
Tripelennamine citrate	8.49	> 100	> 100	Y	
Homochlorcyclizine	8.49	21.13	5.01		
Fluoxetine	8.94	23.71	4.47		Y
Cyclobenzaprine	9.52	14.96	12.59		Y
Chlorprothixene	9.89	23.71	8.91		Y
Bepridil	9.89	> 100	12.59		
Chlorpromazine	10.15	16.79	11.22	Y	Y
Difeterol	11.10	29.85	12.59		
Zotepine	11.10	13.33	12.59		Y
Spiperone	11.99	13.33	12.59	Y	Y
Clemastine fumarate	11.99	23.71	11.22		
Maprotiline	12.30	13.33	12.59		Y
Nylidrin	13.45	26.60	> 100	Y	
Duloxetine	13.97	16.79	8.91		Y
Amoxapine	14.34	5.96	8.91		Y
Clomipramine	14.90	11.88	10.00	Y	Y
Triflupromazine	15.68	23.71	12.59		Y
Bromodiphenhydramine	16.93	N/A	12.59		
Chlormadinone acetate	17.59	> 100	12.59		
Tamoxifen citrate	18.21	26.60	1.78	Y	
Cyclomethycaine	19.00	N/A	12.59		
Bencyclane	19.74	N/A	12.59

### Enrichment of neuroactive drugs in anti-SARS-CoV-2 and AP-1 active compounds

**Figure 4:**
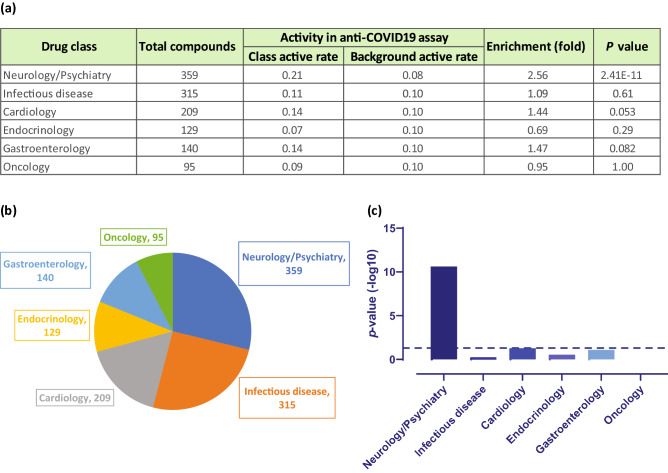
Enrichment of neurology/psychiatry drugs in anti-SARS-CoV-2 active compounds. (a) Activity statistics of six common drug classes in the SARS-CoV-2 CPE assay. Class active rate = Fraction of anti-SARS-CoV-2 active compounds in the drug class; Background active rate = Fraction of anti-SARS-CoV-2 active compounds outside the drug class. (b) Distribution of the six common drug classes in the NPC library. (c) Significance of enrichment of anti-SARS-CoV-2 actives in each drug class. Only the class of neurology/psychiatric drugs was significantly enriched. The dotted line indicates the threshold for statistical significance.

Another interesting phenomenon we observed was that a large number of compounds active in the SARS-CoV-2 CPE assay are psychoactive drugs. We further investigated the statistical significance of this finding. A total of 359 drugs were annotated as neurology/psychiatry drugs and they were tested in the SARS-CoV-2 CPE assay. We found that 74 out of the 359 drugs in this class were active (21%) (Supplementary Table 2; assay activities of example compounds are shown in Fig. 1c), whereas only 8% of drugs that did not belong in this category were active in the SARS-CoV-2 CPE assay. These results corresponded to a 2.6-fold enrichment of actives in the neuroactive drugs and the enrichment was found to be statistically significant (Fisher’s exact test: *P* = 2.41 × 10^–11^). To check whether this phenomenon only occurred in the psychoactive class of drugs, we also examined five other common drugs classes, including infectious disease, cardiology, endocrinology, gastroenterology, and oncology. We found that none of these additional classes were significantly enriched with anti-SARS-CoV-2 active compounds (Fig. 4). The results suggest a possible connection between the psychoactive drugs and the targets/pathways related to SARS-CoV-2 infection or replication in host cells.

## Discussion

In an effort to deconvolute viral targets in pursuit of more effective anti-COVID-19 drug development, we compared the compound activity profiles from our historical qHTS data with recent SARS-CoV-2 CPE assay data. We found that activities against autophagy and AP-1 significantly correlated with anti-SARS-CoV-2 activity. We also found strong associations between the SARS-CoV-2 and other antiviral assays such as SARS-CoV and MERS-CoV pseudo particle entry. It is intuitive given that both SARS-CoV and MERS-CoV are zoonotic beta-coronaviruses with similar genomes and they share a common cellular entry mechanism. Since the emergence of SARS-CoV-2, multiple agents that were shown to be active against MERS-CoV and SARS-CoV over the past 15 years have been tested and continue to exhibit activity against SARS-CoV-2. The analysis conducted in this study using independent assays that were performed years apart reinforces this observation.

Autophagy and endocytosis are interconnected cellular pathways that are involved in the degradation and recycling of intracellular and extracellular components, respectively. The two pathways interact and interdepend on each other, sharing some molecular machinery^39^. The autophagy/endocytosis pathway has been implicated in the entry of coronavirus into host cells, such as hijacking the autophagosomes for viral replication, including SARS-CoV, MERS-CoV and SARS-CoV-2^13,40^. The involvement of autophagy in other coronavirus infections has also been reported^41–43^. Recently, autophagy as a drug target for SARS-CoV-2 has been reviewed in both autophagy and virology journals^40,44,45^. In our recent study, a small number of autophagy modulators were tested to determine if the activity of CQ/HCQ was related to its autophagy modulatory properties, and a number of active compounds were confirmed in the CPE assay^13^. Here, our unbiased comparison of the SARS-CoV-2 CPE assay with approximately 1,000 NCATS qHTS assays targeting various drugs targets and diseases found a significant correlation with an autophagy assay screened against the NPC library several years earlier, further validating our data mining approach. Targeting the autophagy pathway has been tested in clinical trials to curb COVID-19. For example, CQ and HCQ are antimalaria drugs and known autophagy inhibitors. HCQ/CQ have shown promising anti-SARS-CoV-2 activity *in vitro*^13,46,47^; however, their therapeutic effect in COVID-19 patients remain controversial^48,49^. Our analysis here reinforces that more selective and potent modulators of autophagy pathways should be further evaluated in pre-clinical models to test for antiviral activity. Another factor that should be taken into consideration is the mode of entry that the coronavirus can take, either the endosomal or non-endosomal pathway^50^. Blocking autophagy, which is the endocytosis pathway, may not be sufficient to prevent the viral entry, therefore combination approaches, e.g., combination treatments with autophagy inhibitors and TMPRSS2 inhibitors^51^, warrant further consideration.

Activator protein 1 (AP-1) is a dimeric transcription factor composed of proteins belonging to the Jun, Fos, ATF, and JDP families, and it regulates a range of cellular processes. The AP-1 transcription factor family can be activated by different stimuli, such as cytokines, stress, bacterial, and viral infections^52^. The AP-1 signaling pathway has been shown to be activated by the SARS-CoV viral particle^53^, spike protein^54^, nucleocapsid protein^55^, and accessory protein 3b^56^. In a recent study, the AP-1 protein Jun was identified as one of the top hub host proteins, which can either be directly targeted by coronavirus proteins or indirectly involved in the coronavirus infection^57^. The activation of AP-1 signaling may serve as an immune response for the host to fight viral infections. One hypothesis that can be drawn from this observation is that the coronavirus hijacks the AP-1 pathway, which leads to the mediation of the CPE process. Therefore, disruption of the AP-1 pathway could offset this process. While this hypothesis has not been directly tested, the correlation that we found between the AP-1 pathway and SARS-CoV-2 points to a druggable host pathway for SARS-CoV-2 and future emergent coronaviruses. In addition, previous reports have linked the AP-1 pathway to autophagy^58,59^, further validating the correlations between AP-1, autophagy, and SARS-CoV-2.

Psychoactive drugs have been reported to be active against SARS-CoV-2^11,15,19,20^. In this study, we found that the neurology/psychiatry class of drugs, in contrast to other classes of drugs, was significantly enriched in anti-SARS-CoV-2 activity. Most of the active compounds in the neurology/psychiatry class of drugs are psychoactive drugs which target G protein coupled receptors (GPCRs), particularly monoamine receptors (86% of the psychoactive drugs that showed anti-SARS-CoV-2 activity belong to this category) (Supplementary Table 2). We hypothesize that those compounds may bind to membrane receptors and activate intracellular pathways to fight against coronaviruses. It is interesting to note that among the active compounds in both the AP-1 and SARS-CoV-2 CPE assays, we found a more pronounced enrichment of neurology/psychiatry drugs (3.76-fold; *P* = 3.89 × 10^–11^), suggesting that this class of drugs may also act through the AP-1 pathway to inhibit SARS-CoV-2. Another hypothesis is that SARS-CoV-2 may infect cells through other unknown membrane proteins in addition to angiotensin-converting enzyme 2 (ACE2), and those compounds may interfere with the viral binding to its receptors. GPCRs have been shown to be hijacked by viruses as co-receptors for entry into host cells^60–62^. A French study reported that lower incidences of the symptomatic forms of COVID-19 were found among psychiatric patients (~ 4%) when compared to clinical staff (~ 14%)^63^. An anti-psychiatric drug, chlorpromazine (Fig. 3), has been repurposed for COVID-19 treatment, and it is currently in phase III clinical trial (https://clinicaltrials.gov/ct2/show/NCT04366739). In another phase II clinical trial, fluvoxamine, a selective serotonin reuptake inhibitor (SSRI), was found to prevent more serious complications of COVID-19 infection (https://clinicaltrials.gov/ct2/show/NCT04342663). Pre-clinical data and clinical observations of those psychoactive drugs are promising; however, further clinical evidence and data are required to confirm the anti-SARS-CoV-2 effects of those psychoactive drugs.

In summary, we discovered that the autophagy and AP-1 signaling pathways may be potential targets for COVID-19 therapeutics through systematic mining of a large qHTS database. These findings from in vitro experiments need to be further validated by in vivo animal studies and human clinical trials to confirm the therapeutic efficacy of the identified targets. In addition, the class of neurology/psychiatry drugs was found to be significantly enriched with anti-SARS-CoV-2 active compounds, indicating that this class of drugs has the potential to be repurposed as treatments for COVID-19 and warrant further investigation.

## Materials and methods

### SARS-CoV-2 cytopathic effect (CPE) assay

Vero-E6 cells previously selected for high ACE2 expression^64^ (grown in EMEM, 10% FBS, and 1% Penicillin/Streptomycin) were cultured in T175 flasks and passaged at 95% confluency. Cells were washed once with PBS and dissociated from the flask using TrypLE. Cells were counted prior to seeding. A CPE assay previously used to measure antiviral effects against SARS-CoV^65^ was adapted for performance in 384 well plates to measure CPE of SARS CoV-2 with the following modifications. Cells, harvested and suspended at 160,000 cells/ml in MEM/1% PSG/1% HEPES supplemented 2% HI FBS, were batch inoculated with SARS CoV-2 (USA_WA1/2020) at M.O.I. of approximately 0.002 which resulted in approximately 5% cell viability 72 h post infection. Compound solutions in DMSO were acoustically dispensed into assay ready plates (ARPs) at 3 point 1:5 titrations. ARPs were stored at -20 °C and shipped to BSL3 facility (Southern Research Institute, Birmingham, AL) for CPE assay. ARPs were brought to room temperature and 5 µl of assay media was dispensed to all wells. The plates were transported into the BSL-3 facility were a 25 μL aliquot of virus inoculated cells (4000 Vero E6 cells/well) was added to each well in columns 3–24. The wells in columns 23–24 contained virus infected cells only (no compound treatment). A 25 μL aliquot of uninfected cells was added to columns 1–2 of each plate for the cell only (no virus) controls. After incubating plates at 37 °C with 5% CO_2_ and 90% humidity for 72 h, 30 μL of Cell Titer-Glo (Promega, Madison, WI) was added to each well. Following incubation at room temperature for 10 min the plates were sealed with a clear cover, surface decontaminated, and luminescence was read using a Perkin Elmer Envision (Waltham, MA) plate reader to measure cell viability.

### AP-1-bla ME-180 assay

CellSensor AP-1-bla ME-180 cell line and the culture medium components were purchased from Thermo Fisher Scientific (Waltham, MA). These cells contain a beta-lactamase reporter gene under the control of AP-1 response element that has been stably integrated into ME-180 cells. Cells were cultured in DMEM medium supplemented with 10% dialyzed fetal bovine serum (FBS), 0.1 mM non-essential amino acids (NEAA), 1 mM sodium pyruvate, 25 mM HEPES, 100 U/ml penicillin, 100 μg/ml streptomycin, and 5 μg/ml of blastcidin at 37 °C under a humidified atmosphere and 5% CO_2_. AP-1-bla ME-180 cells were used to screen the NPC compound collection. The positive controls, human epidermal growth factor (EGF) for the AP-1-bla and tetraoctyl ammonium bromide for the cytotoxicity assays, were purchased from Sigma-Aldrich (St. Louis, MO).

CellSensor AP-1-bla ME-180 cells were suspended in 6 μL of assay medium (Opti-MEM with 0.5% dialyzed FBS, 0.1 mM NEAA, 1 mM sodium pyruvate, 100 U/ml penicillin, and 100 μg/ml streptomycin), and were dispensed at 2,500 cells per well in 1,536-well tissue culture treated black/clear bottom plates (Greiner Bio-One North America, NC) using a Multidrop Combi (Thermo Fisher Scientific). After incubation at 37 °C for an overnight to facilitate cell adhesion, 23 nL of compounds and positive controls were transferred into the assay plates by a Pintool station (Wako Automation, San Diego, CA). The assay plates were incubated for 5 h at 37 °C. One μl of LiveBLAzer FRET B/G (CCF4-AM) substrate mix (Thermo Fisher Scientific) was added using an Flying Reagent Dispenser (FRD) and incubated at room temperature for 2 h. The fluorescence signal was measured using an Envision plate reader (Perkin Elmer, Waltham, MA) at excitation 405 nm, and dual emissions at 460 and 530 nm. Data were expressed as relative fluorescence units (ratio of 460 nm/530 nm emissions). The cytotoxicity of each compound was tested in parallel in the same well by adding 4 µl/well of CellTiter-Glo reagent (Promega, Madison, WI) after beta-lactamase read into the assay plates using an FRD. After 30 min incubation at room temperature, the luminescence signal was measured using a ViewLux plate reader (Perkin Elmer). Cytotoxicity data were expressed as relative luminescence units.

## Supplementary Information

Supplementary Information
