# Supplementary material for: Mining of high throughput screening database reveals AP-1 and autophagy pathways as potential targets for COVID-19 therapeutics
Source: Sci Rep. 2021 Mar 24;11:6725. doi: 10.1038/s41598-021-86110-8 (PMC7990955; doi:10.1038/s41598-021-86110-8)
Supplement: Supplementary file 1 — Supplementary Information [file 41598_2021_86110_MOESM1_ESM.pdf]

Supplementary Information

Mining of high throughput screening database reveals AP-1 and autophagy pathways as potential targets for COVID-19 therapeutics

Hu Zhu,[1] Catherine Z. Chen[1], Srilatha Sakamuru[1], Jinghua Zhao,[1] Deborah Ngan,[1] Anton Simeonov,[1] Mathew D. Hall,[1] Menghang Xia[1], Wei Zheng[1], Ruili Huang[1*]

[1]Division of Preclinical Innovation, National Center for Advancing Translational Sciences (NCATS), National Institutes of Health (NIH), Rockville, MD 20850, USA.

*Address correspondence and reprint requests to

Ruili Huang, Ph.D.

9800 Medical Center Drive

DPI/NCATS

National Institutes of Health

Rockville, MD 20850

Phone: 301-827-0944

Fax: 301-217-5736

Email: huangru@mail.nih.gov

Table S1. Compounds with reported anti-SARS-CoV-2 activity.

Name
(+)-Pagoclone
(1-[(4-Chlorophenyl)phenyl-methyl]-4-methylpiperazine)
(S)-Propranolol hydrochloride
11?-Prostaglandin E1
2,6-Diiodo-4-nitrophenol
8-iso Misoprostol
9-Amino-1,2,3,4-tetrahydroacridine hydrochloride
Abemaciclib
ABT-333
Acetopromazine maleate
Acitretin
Albuterol hemisulfate
ALVERINE CITRATE
Ambrisentan
Ambroxol hydrochloride
Amiodarone hydrochloride
Amitriptyline hydrochloride
Amlodipine besylate
Amodiaquin dihydrochloride dihydrate
Amoxapine
Anethole trithione
Anidulafungin (LY303366)
Anthralin
Apilimod
Aprindine hydrochloride
Arbidol
Arotinoid acid
Artenimol
Astemizole
Azelastine hydrochloride
Azithromycin
Benazepril Hydrochloride
Bencyclane
Benoxinate hydrochloride
Benztropine mesylate
Benzydamine hydrochloride
Bepridil hydrochloride
Bexarotene
BF 2649 hydrochloride

Bifemelane
Bisbentiamine
Blonanserin
Brexpiprazole
Bromodiphenhydramine
Buclizine HCl
Budralazine
Bunamidine hydrochloride
Buspirone hydrochloride
Cabergoline
Candesartan
Carbetapentane citrate
Carmofur
Carvedilol
CEFAMANDOLE SODIUM
Celecoxib
Cephaeline dihydrochloride heptahydrate
Cepharanthine
Ceritinib
Cetilistat
Chlormadinone acetate
Chloroquine diphosphate
Chloroxine
Chlorpromazine hydrochloride
Chlorprothixene
Ciclesonide
CICLOPIROX OLAMINE
Cilnidipine
Cisatracurium besylate
CLEBOPRIDE MALEATE
Clemastine fumarate
Clemizol
Clioquinol
Clofazimine
Clomipramine hydrochloride
Cloperastine hydrochloride
Closantel Sodium
Clozapine
Cyclobenzaprine hydrochloride
Cyclofenil
Cyclomethycaine
CYCLOPENTOLATE HYDROCHLORIDE
Cyproheptadine hydrochloride

Desonide
Danazol
Dapoxetine HCl
Darunavir
Darunavir
Deferasirox
Deserpidine
Desipramine hydrochloride
Desloratadine
Desogestrel
DIBUCAINE HYDROCHLORIDE
Dicyclomine hydrochloride
Diethylstilbestrol
Difeterol
Dimaprit dihydrochloride
Dimethisoquin hydrochloride
Diphenhydramine hydrochloride
Dipivefrin hydrochloride
Dipyrithione
DL-Cycloserine
Dolutegravir
Donepezil hydrochloride
Dopamine hydrochloride
Doramectin
Dothiepin hydrochloride
Doxazosin mesylate
Duloxetine HCl
DYCLONINE HYDROCHLORIDE
Ebselen
Eletriptan hydrobromide
Eliglustat (Tartrate)
Eltrombopag Olamine
Emetine dihydrochloride hydrate
Emodepside
ENOXACIN
Enzastaurin
Eperisone hydrochloride
Equilin
Ethopropazine hydrochloride
ETHOXZOLAMIDE
Ethoxzolamide
Etifelmine hydrochloride
Etiocholan-3Î±-ol-17-one

Exemestane
Famotidine
Fenoldopam bromide
Flunarizine dihydrochloride
Fluoxetine hydrochloride
Fluphenazine decanoate dihydrochloride
Fluphenazine dihydrochloride
Fluralaner
Fluspirilene
Fonazine
Formestane
Formoterol Fumarate
Fosinopril sodium
Fursultiamine
Halofantrine
Haloperidol (D4)
Hexachlorophene
HEXETIDINE
HOMOCHLORCYCLIZINE
Homoharringtonine
HYCANTHONE
Hydroquinine
Hydroxychloroquine sulfate
Hydroxyprogesterone caproate
Ibudilast
Idazoxan hydrochloride
Ifenprodil hemitartrate
Iloperidone
Imatinib
Imipramine hydrochloride
InterBioScreen,BB_SC-1871
IODOQUINOL
Isotretinoin
Itraconazole
Ivermectin
Ketanserin tartrate
Labetalol hydrochloride
Lapatinib
LASALOCID SODIUM
Lemildipine
Lercanidipine hydrochloride hemihydrate
L-Glutathione, oxidized (sodium salt)
Lomerizine dihydrochloride

Loperamide hydrochloride
Lopinavir
Loratadine
LOSARTAN
Loxapine succinate
Lufenuron
Lycorine
Lynestrenol
Manidipine
Maprotiline hydrochloride
Maropitant
MDL 28170
Mefloquine hydrochloride
Melitracen hydrochloride
Meprylcaine hydrochloride
Meptazinol hydrochloride
Mesoridazine Besylate
Methandrostenolone
Methdilazine Hydrochloride
Methotrimeprazine maleat salt
Metixene hydrochloride
Mirtazapine
MK-4827 Racemate
Mometasone furoate
Monatepil
N,N'-Dibenzylethane-1,2-diamine dihydrochloride
NABUMETONE
Nafronyl oxalate
Naftopidil dihydrochloride
Nalmefene
Narasin
Nelfinavir
Nelfinavir mesylate
Nicardipine hydrochloride
Niclosamide
Nitazoxanide
NKH477
Nortriptyline hydrochloride
Nylidrin hydrochloride
Ofloxacin
Olanzapine
Olmesartan
Omeprazole

Omoconazole
Ondansetron hydrochloride
Opipramol dihydrochloride
Orphenadrine hydrochloride
Ospemifene
Oxatomide
Oxprenolol hydrochloride
Oxybutynin Chloride
Oxymetholone
Oxyphenisatin
Pergolide mesylate
Periciazine
Perphenazine (D8 Dihydrochloride)
PHENTERMINE
Piperacetazine
Piroctone olamine
Pizotifen
Posaconazole
PREDNICARBATE
Pregnenolone
Prochlorperazine dimaleate
Proglumetacin
Promazine hydrochloride
Promethazine hydrochloride
Propafenone hydrochloride
Propionylpromazine hydrochloride
Propiverine hydrochloride
Protionamide
Protriptyline hydrochloride
P-T-BUTYL-M-CRESOL
PX-12
Pyrimethamine
Quinine sulfate
Raloxifene hydrochloride
Ranolazine dihydrochloride
Rescimetol
RESERPINE
Retapamulin
Ribavirin
Ritonavir
Rupatadine Fumarate
Sarpogrelate
Shikonin

Siccanin
SNX-2112 (PF-04928473)
Sodium bithionolate
Sofalcone
Sorafenib
SPARFLOXACIN
Spiclomazine hydrochloride
Spiperone hydrochloride
Spiramycin II
Sulfadoxine
Sulfadoxine
T-705(Favipiravir)
TAK 438
Tamibarotene
Tamoxifen citrate
Tazarotene
TDZD-8
Teicoplanin
Terconazole
Tetraethylthiuram disulfide
TETRANDRINE
Thiethylperazine malate
Thiethylperazine malate
Thioproperazine dimethanesulfonate
Thiothixene hydrochloride
Tideglusib
Tilorone
Timiperone
Tolperisone hydrochloride
Tolterodine
Toremifene Citrate
Triamterene
Tribromsalan
Trifluomeprazine 2-butenedioate
Triflupromazine hydrochloride
Trihexyphenidyl hydrochloride
Trimeprazine tartrate
Trimetrexate
Trimipramine maleate
Triparanol
Tripelennamine citrate
TYLOXAPOL
VBY-825

VILAZODONE
Vorapaxar Sulfate
Xylometazoline hydrochloride
Zaleplon
Zanapezil fumerate
ZK 93426 hydrochloride
Zoledronic acid monohydrate
Zotepine

Table S2. Neurology/psychiatry drugs active in the SARS-CoV-2 CPE assay.

Name	MoA
Benztropine mesylate	acetylcholine receptor antagonist
Cisatracurium besylate	acetylcholine receptor antagonist
Eperisone	acetylcholine receptor antagonist
Orphenadrine	acetylcholine receptor antagonist
Trihexyphenidyl	acetylcholine receptor antagonist
Bifemelane	acetylcholine release enhancer
9-Amino-1,2,3,4-tetrahydroacridine	acetylcholinesterase inhibitor
Donepezil	acetylcholinesterase inhibitor
Cyclobenzaprine	adrenergic receptor agonist; serotonin receptor agonist
(S)-Propranolol	adrenergic receptor antagonist
Spiclomazine	adrenergic receptor antagonist
Trifluomeprazine 2-butenedioate	adrenergic receptor antagonist; dopamine receptor antagonist
Mirtazapine	adrenergic receptor antagonist; serotonin receptor antagonist
Tetraethylthiuram disulfide	aldehyde dehydrogenase inhibitor; DNA methyltransferase inhibitor; TRPV agonist
Zaleplon	benzodiazepine receptor agonist
Flunarizine	calcium channel blocker
Lomerizine	calcium channel blocker
Ethopropazine	constitutive androstane receptor (CAR) agonist
Proglumetacin	cyclooxygenase inhibitor
Pergolide mesylate	dopamine D1-receptor agonist; dopamine D2-receptor agonist
PERICIAZINE	dopamine D2-receptor antagonist
Timiperone	dopamine D2-receptor antagonist
Propionylpromazine	dopamine D2-receptor antagonist; trypanothione reductase inhibitor
Cabergoline	dopamine receptor agonist
Dopamine	dopamine receptor agonist
Fenoldopam bromide	dopamine receptor agonist

Chlorpromazine	dopamine receptor antagonist
Chlorprothixene	dopamine receptor antagonist
CLEBOPRIDE MALEATE	dopamine receptor antagonist
Fluphenazine	dopamine receptor antagonist
Fluspirilene	dopamine receptor antagonist
Haloperidol	dopamine receptor antagonist
Mesoridazine Besylate	dopamine receptor antagonist
Perphenazine	dopamine receptor antagonist
Piperacetazine	dopamine receptor antagonist
Prochlorperazine dimaleate	dopamine receptor antagonist
Promazine	dopamine receptor antagonist
Spiperone	dopamine receptor antagonist
Thioproperazine dimethanesulfonate	dopamine receptor antagonist
Thiothixene	dopamine receptor antagonist
Triflupromazine	dopamine receptor antagonist
Iloperidone	dopamine receptor antagonist; serotonin receptor antagonist
Loxapine succinate	dopamine receptor antagonist; serotonin receptor antagonist
Olanzapine (LY170053)	dopamine receptor antagonist; serotonin receptor antagonist
Zotepine	dopamine receptor antagonist; serotonin receptor antagonist
Formoterol	glutamate receptor antagonist
Buclizine	histamine receptor antagonist
Diphenhydramine	histamine receptor antagonist
Loratadine	histamine receptor antagonist
Promethazine	histamine receptor antagonist
Trimeprazine tartrate	histamine receptor ligand; histamine receptor antagonist
Ibudilast	leukotriene receptor antagonist; phosphodiesterase inhibitor
Dimethisoquin	local anesthetic
Amitriptyline	norepinephrine inhibitor; norepinephrine reuptake inhibitor; serotonin receptor antagonist; serotonin?norepinephrine reuptake inhibitor (SNRI)
Amoxapine	norepinephrine reputake inhibitor
Imipramine	norepinephrine reputake inhibitor; serotonin reuptake inhibitor
Maprotiline	norepinephrine reputake inhibitor; tricyclic antidepressant
Trimipramine maleate	norepinephrine reputake inhibitor; tricyclic antidepressant
Duloxetine	norepinephrine reuptake inhibitor; serotonin?norepinephrine reuptake inhibitor (SNRI)
Dosulepin	norepinephrine reuptake inhibitor; serotonin?norepinephrine reuptake inhibitor (SNRI); tricyclic antidepressant
Meptazinol	opioid receptor agonist
PREDNICARBATE	phospholipase activator
Fluoxetine	selective serotonin reuptake inhibitor (SSRI)
Buspirone	serotonin receptor agonist
Eletriptan	serotonin receptor agonist

Pizotifen	serotonin receptor antagonist
Clomipramine	serotonin transporter (SERT) inhibitor
Opipramol	sigma receptor agonist
DIBUCAINE	sodium channel blocker
DYCLONINE	sodium channel blocker
Desipramine	tricyclic antidepressant
Nortriptyline	tricyclic antidepressant
Protriptyline	tricyclic antidepressant
Tolperisone	voltage-gated sodium channel blocker
